# Radioscopic Examination of the Lungs and Its Limited Sensitiveness

**Published:** 1919

**Authors:** 


***Radioscopic Examination of the Lungs and its Limited Sensitiveness.*** By MM. CH. MANTOUX and G. MAINGOT. *Bulletin de la Société Médicale des Hôpitaux de Paris*, October 24, 1918.

In the immense majority of clinical examinations of the thorax, auscultation and radiologic researches give on the whole quite comparable results.

Nevertheless, in some cases the diagnosis based upon both methods is not comparable. If the lesions are deeply located and separated from the ear by a deep layer of sound pulmonary tissues, they may be detected only by X-rays, while on the contrary some lesions perceivable with the ear are invisible with X-rays. Such are lesions developing in bronchitis and dry pleuritis and presenting no concomitant inspissating of the pulmonary tissues; lastly there are some tuberculous infiltrating lesions invisible on the radioscopic screen because of insufficient sensitiveness of the method.

Very small lesions are undetected by X-rays, as are the very fine details of bone structure. Below a certain extent and a certain thickness, the foci of tuberculosis infiltration are invisible on the screen.

The authors have determined experimentally the conditions of visibility of tuberculous lesions. The tests were undertaken with normal subjects of normal stoutness, and fragments of tuberculous tissues removed at necropsies.

The following results were obtained :

Fragments of tissues, 5 to 7 mm. thick and extending over several cm. square, gave shadows barely visible on the screen, as did also a fragment of infiltrated pleura, 15 mm. thick. A distinct shadow was obtained only with a fragment of ganglion 2 cm. thick.

None of these fragments gave visible shadows if examined through a stout person.

Thus the authors conclude that the non-detection of tuberculous lesions after a radioscopic examination does not prove with certainty that there are no pulmonary lesions or pleural alterations.

